# Establishment and genetic characterization of a novel mixed-phenotype acute leukemia cell line with EP300-ZNF384 fusion

**DOI:** 10.1186/s13045-015-0197-2

**Published:** 2015-08-21

**Authors:** Nana Ping, Huiying Qiu, Qian Wang, Haiping Dai, Changgeng Ruan, Stefan Ehrentraut, Hans G. Drexler, Roderick A. F. MacLeod, Suning Chen

**Affiliations:** Jiangsu Institute of Hematology, Key Laboratory of Thrombosis and Hemostasis of Ministry of Health, The First Affiliated Hospital of Soochow University, Suzhou, People’s Republic of China; DSMZ - German Collection of Microorganisms and Cell Cultures, Braunschweig, Germany; Collaborative Innovation Center of Hematology, Soochow University, Suzhou, People’s Republic of China

**Keywords:** Mixed-phenotype acute leukemia, Leukemia cell line, Next-generation sequencing, EP300-ZNF384

## Abstract

Herein, we describe the establishment and characterization of the first mixed-phenotype acute leukemia cell line (JIH-5). The JIH-5 cell line was established from leukemia cells with B lymphoid/myeloid phenotype from a female mixed-phenotype acute leukemia patient. JIH-5 cells exhibit an immunophenotype comprised of myeloid and B lymphoid antigens. Whole-exome sequencing revealed somatic mutations in nine genes in JIH-5 cells. Transcriptional sequencing of JIH-5 cells identified EP300-ZNF384 fusion transcript, which is a recurrent alteration in B cell acute lymphoblastic leukemia. Our results suggest that the JIH-5 cell line may serve as a tool for the study of mixed-phenotype acute leukemia or EP300-ZNF384.

## Findings

In a minority of patients with acute leukemia, it is difficult to determine the lineage origin because of the expression of both lymphoid and myeloid lineage-specific antigens [1–5]. The 2008 World Health Organization (WHO) classification introduced a new designation for this entity, mixed-phenotype acute leukemia (MPAL) [6]. Tumor cells are characterized by various biomarkers, such as cytogenetic, molecular genetic, or epigenetic aberrations [7–11]. However, the pathogenesis and optimal therapy of patients with MPAL remain largely undefined. Although several leukemia cell lines were once reported as BAL cell lines [12–17], none fulfill the WHO 2008 criteria for MPAL. So far, no cell line established from patients with MPAL has been reported. Recently, we established the first human MPAL cell line, JIH-5. Herein, we present the phenotypic, genetic, and functional properties of JIH-5 cells. We applied next-generation sequencing (NGS) technology to unravel the transcriptome of JIH-5 cells.

A 21-year-old female with MPAL was admitted to our hospital in December 2008. Bone marrow sample was obtained from the patient with informed consent in December 2009 during the second relapse. Mononuclear cells were cultured in Iscove’s Modified Dulbecco’s Medium with 20 % fetal calf serum. The leukemia cells exhibited gradual cell proliferation 2 months after primary culture was initiated. The cell line was designated JIH-5. JIH-5 cells were tolerant to freezing in defined medium, storage in liquid nitrogen, thawing, and subsequent expansion. JIH-5 cells grow as single cells in suspension culture.

**Fig. 1:**
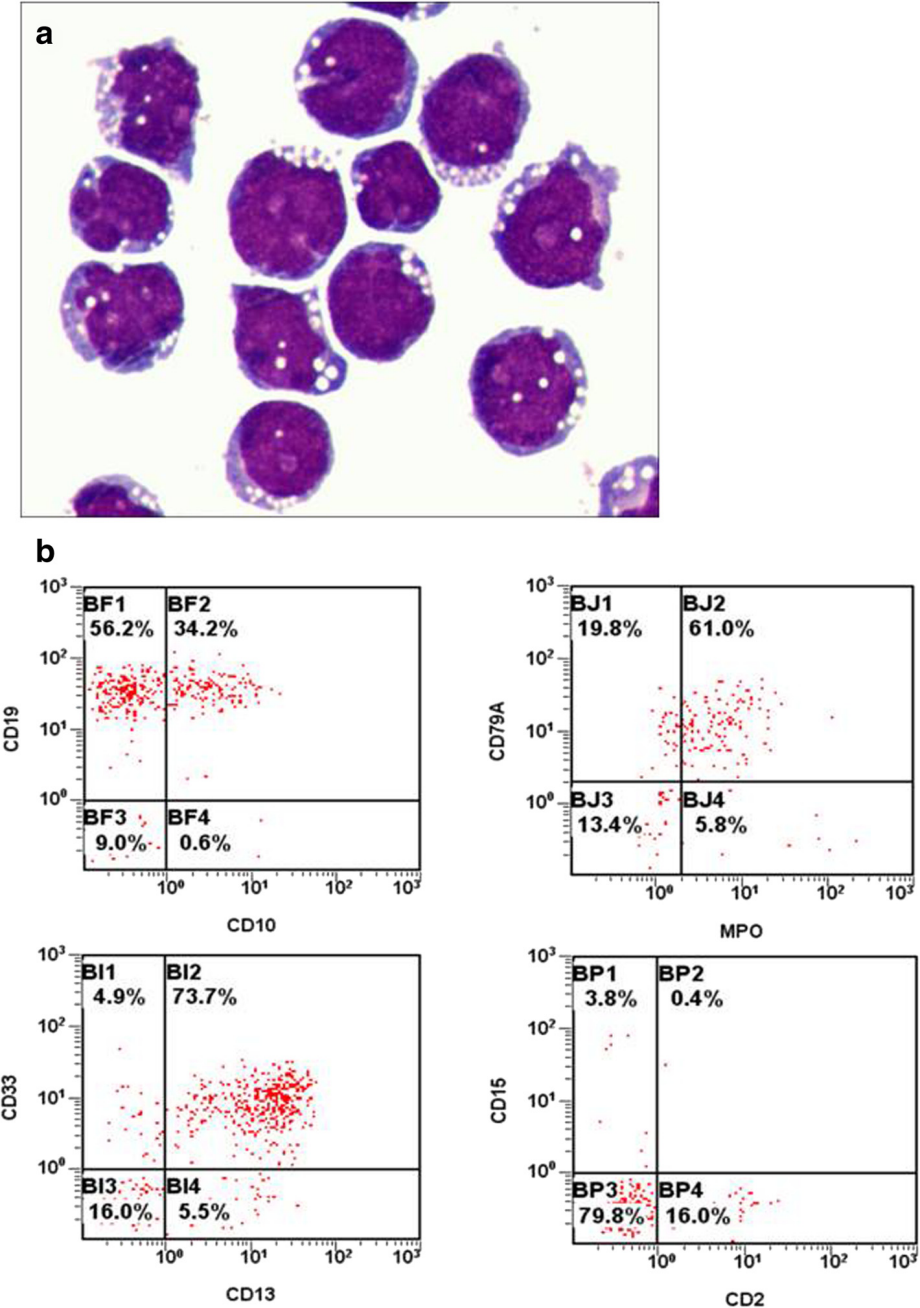
Morphological and immunophenotypic analysis of JIH-5. **a**. Morphology of SHI-1 cells on Wright’s staining under a light microscopy (original magnification ×1000). **b**. Immunophenotypic features of JIH-5 cells

JIH-5 cells exhibit medium-sized spheroidal morphologies and large round nuclei with fine nuclear chromatin (Fig. 1a). The immunoprofiles of the JIH-5 cells are summarized in Table 1. JIH-5 cells express typical antigens of myeloid lineages (CD13, CD15, CD33, and cMPO), as well as antigens of B lymphoid lineages (CD10, CD19, CD22, CD23, and cCD79a) (Fig. 1b). The negative polymerase chain reaction (PCR) results with EBV and mycoplasma specific primers excluded EBV and mycoplasma contamination. The colony formation rate of JIH-5 cells was 1.41 % by semi-solid methylcellulose clonogenic assay. Tumor masses were found in one of six mice injected with JIH-5 cells after 83 days. The genetic identity of JIH-5 cells was compared to BM cell sample from the patient using short tandem repeat PCR. The results of authentication analysis indicated that the JIH-5 cells remained genetically identical to the founding tumor cells.

**Table 1:** Immunophenotypic characterization of the JIH-5 cells and the primary leukemia cells

Antigen (CD)	Primary leukemia cells (%)		JIH-5 cells (%)
	At presentation	At the second relapse	
T/NK cell markers			
CD2	0.2	0.5	16.4
CD3	ND	ND	1.1
CD5	ND	ND	1.1
CD7	0.3	0.6	0
CD56	ND	ND	1.4
cCD3	ND	0	0.3
B cell markers			
CD10	5.6	11.6	34.8
CD19	99.8	93.9	90.4
CD20	0.1	0.5	1.7
CD22	ND	ND	73.6
CD23	ND	ND	53.3
FMC-7	ND	ND	2.1
cCD79a	97.6	93.6	80.8
Myeloid markers			
CD13	28.9	72.2	79.2
CD14	0.2	1.0	2.1
CD15	14.1	7.3	4.2
CD33	85.2	80.9	78.6
CD64	ND	ND	5.6
MPO	11.9	59.1	66.8
Progenitor markers			
CD34	92.5	95.3	13.6
CD38	ND	ND	30
CD117	0.1	0.8	3.6
HLA-DR	65.8	11.2	52.2
Adhesion markers			
CD11b	ND	ND	0.9
Erythroid markers			
CD71	ND	ND	5.3
GPA	ND	ND	1.3
Megakaryocytic markers			
CD41	ND	ND	47.2
CD61	ND	ND	1.1
Plasma cell markers			
CD138	ND	ND	1.8

*ND* not done

**Fig. 2:**
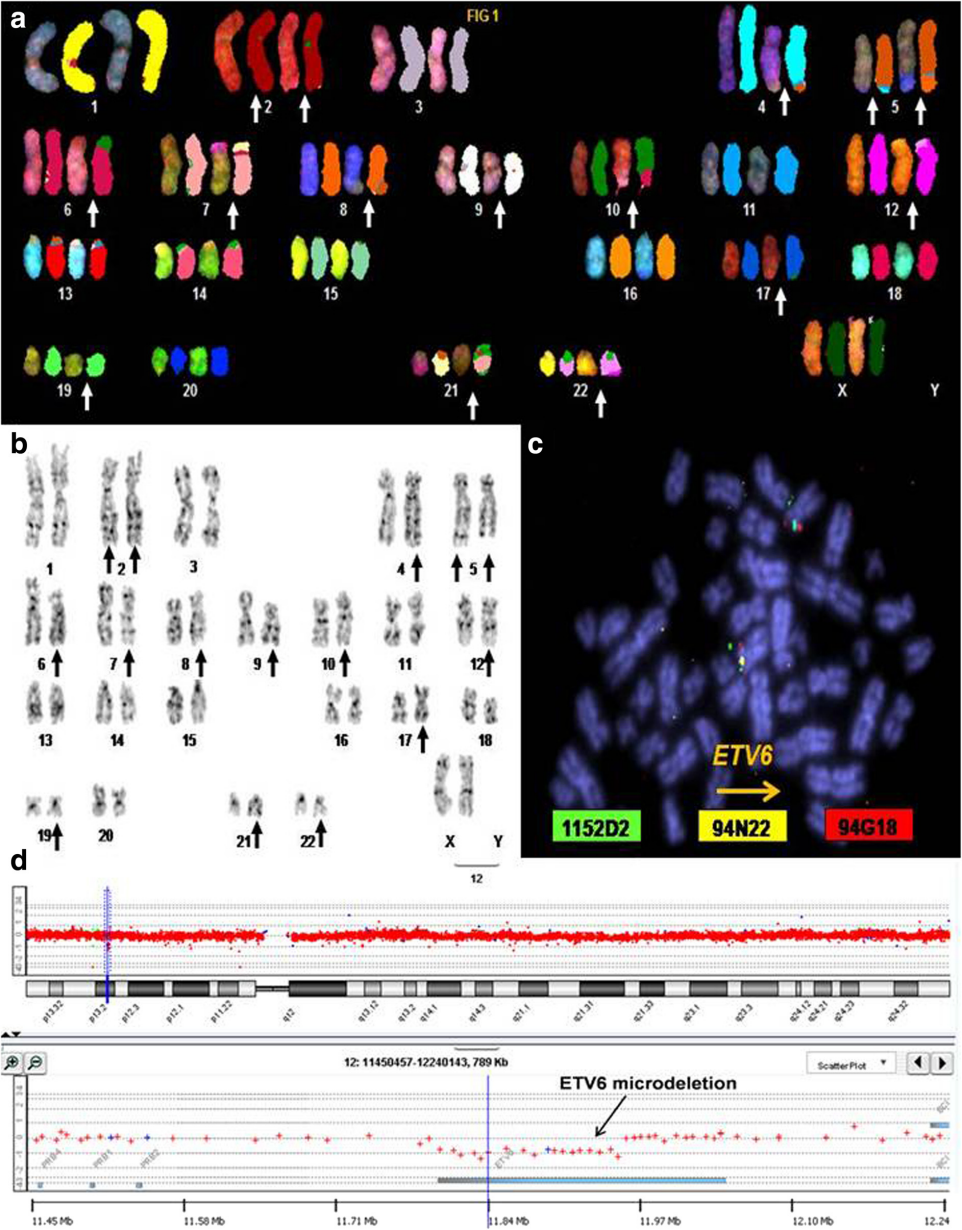
Cytogenetic analysis of JIH-5. Analysis by SKY (**a**) and G-banding (**b**) revealed a complex pseudodiploid karyotype in which 15/46 chromosomes showed visible rearrangements (*arrows*). FISH analysis using golden path clones of genes at/near breakpoints identified a microdeletion affecting the 12p13 region encompassing BAC clone RP11-94N22 which bears the ETV6 gene (**c**). **d** Image shows array CGH (244 K) analysis in JIH-5 cells revealed a 0.15-Mb deletion (11.80–11.95 Mb) in the ETV6 gene. Cytogenetic harvesting, labeling, and fluorescence microscopy were performed as described previously

Combined G-banding and spectral karyotype (SKY) yielded the following karyotype for JIH-5: 46,XX,del(2)(q33)t(2;2)(p22;q37),t(4;5)(q35;q35),t(5;8)(q32;q22),der(6)del(6)(p21p22)t(6;10)(p23;q23),t(7;21)(p15;q21),der(9)del(9)(p21)del(9)(q34.2),der(10)t(6;10),t(12;22)(p13;q13),der(17)t(17;17)(p13;q22),del(19)(q13) (Fig. 2a, b). A total of ten copy number alterations (CNA) were detected by a-CGH. Both fluorescence in situ hybridization (FISH) and a-CGH analysis showed a microdeletion affecting ETV6 gene (Fig. 2c, d). No mutations were detected in 15 acute leukemia-related genes by direct sequencing of PCR products in JIH-5 cells. The global expression profile of JIH-5 was compared to leukemic blast cells from the patient, and a range of cell lines representing B and T cell acute lymphoblastic leukemia (T-ALL). The results indicate that transcriptionally, JIH-5 cells more closely resemble cell lines of B rather than T-ALL origin.

**Fig. 3:**
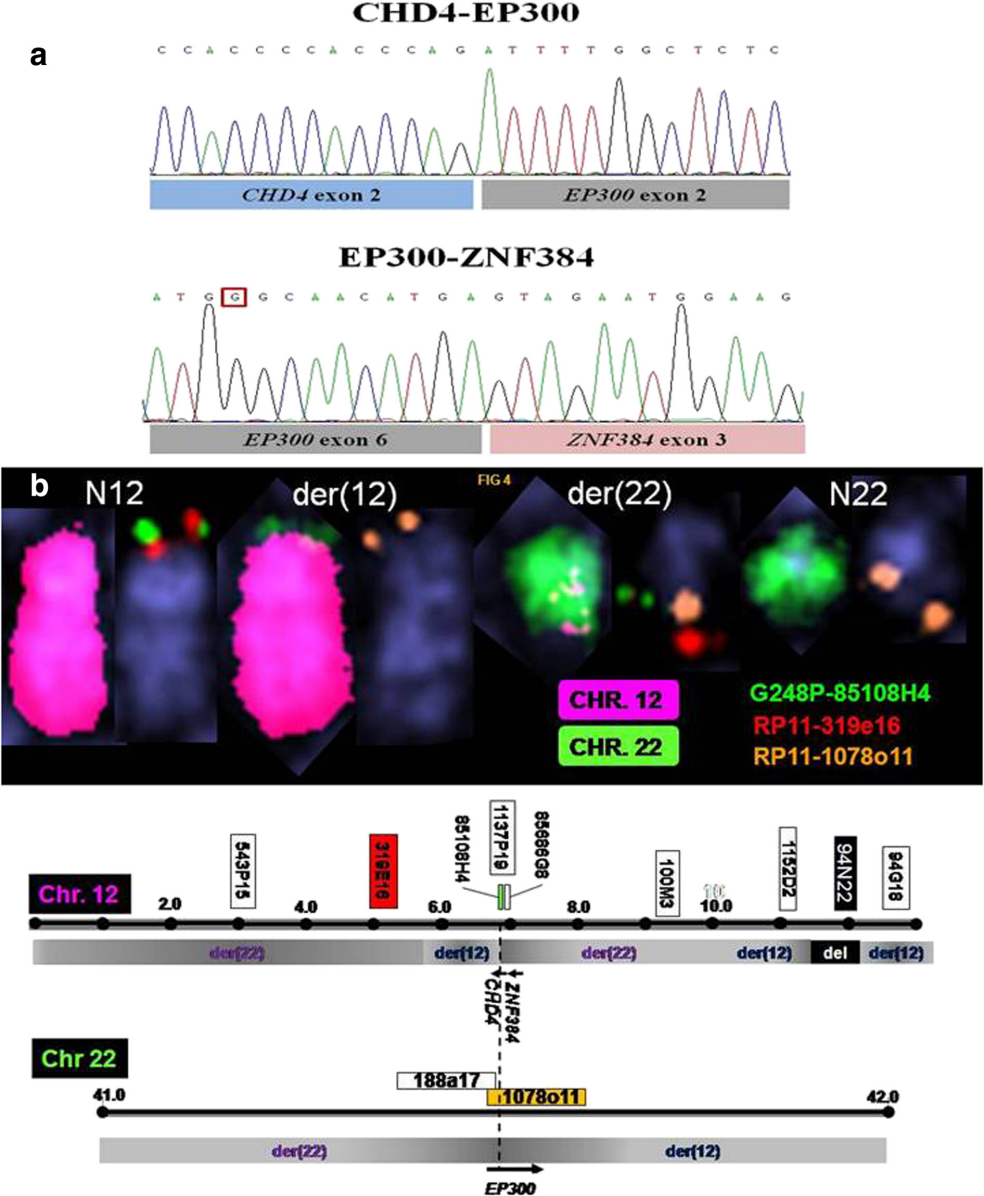
Fusion of EP300 (located at 22q13) with both CHD4 and ZNF384 (at 12p13). **a** Sanger sequencing data confirmed two novel fusion transcripts involving EP300 gene, involving CHD4 (exon 2) with EP300 (exon 2) and EP300 (exon 6) with ZNF384 (exon 3). **b** Fusion of EP300 (located at 22q13) with both CHD4 and ZNF384 (at 12p13) appears to have resulted from a complex series of genomic rearrangements as shown by chromosome painting (*left homologues*) and FISH (*right*) using tilepath BAC and fosmid clones (*upper panel*). Note the presence of two discrete regions of chr. 12-derived material on the der(22) implying a complex, possibly insertional, event. This picture is supported by FISH revealing interspersal of chr. 12- and 22-derived BAC clones over circa 9 MBp from 12p13 (*lower panel*). FISH revealed breakpoints within RP11-1137p19 and 1078o11 involving the ZNF384/CHD4 and EP300 regions implicated in fusion events

We captured and sequenced exomes from the paired sample of JIH-5 cells and control specimen in remission. We detected somatic tumor-specific mutations in a total of nine genes (eight missense and one nonsense mutations), including ABCA8, BCHE, CALCA, CSTF2, FPR1, KCNJ8, MAFB, STMN1, and TAAR8; all were heterozygous in JIH-5 cells. Bioinformatic evaluation of the transcriptional sequencing data and RT-PCR verification revealed six novel fusions, comprising three acting as translocations: EP300 (at 22q13) with both the adjacent ZNF384 and CHD4 (12p13), MSH2 (2p21) with NLK (17q11), and three microdeletions, HACL1-COLQ (3p25), HDAC8-CITED1 (Xq13), and POLA2-CDC42EP2 (11q13). Interestingly, the EP300 gene was found to fuse simultaneously with two partner genes located in 12p13, CHD4, and ZNF384 (Fig. 3a). Further FISH analysis with BAC and fosmid clones flanking EP300, CHD4, and ZNF384 confirmed breakpoints within CHD4 and EP300 due to a complex, apparently insertional, rearrangement involving 12p13 and 22q13 (Fig. 3b). Mutations of EP300 have been detected in Rubinstein-Taybi syndrome and some solid tumors [18–22]. The EP300 was found to be fused with MLL in an AML patient harboring t(11;22)(q23;q13) [23]. CHD4 encodes a catalytic subunit of the NuRD complex and plays an important role in transcriptional regulation, chromatin assembly, and DNA damage repair [24]. The ZNF384 gene has been observed recurrently fused with EWSR1, TAF15, or E2A in acute leukemia [25, 26]. Recently, the EP300-ZNF384 was identified as a recurrent aberration in B cell acute lymphoblastic leukemia (B-ALL) [27]. The genetic abnormalities found in JIH-5 cells are detailed in Table 2.

**Table 2:** Synopsis of data on the JIH-5 cell line

Parameter	JIH-5
Clinical data	
Patient	21-year-old female
Diagnosis	MPAL
Treatment status	At the second relapse
Specimen	BM
Year of establishment	2009
Culture characterization	
Culture medium	IMDM + 20 % FCS
Growth pattern	Single cells in suspension
Doubling time	97 h
Optimal cell density	1 × 10^6^cells/ml
Optimal split	1:3 every 3–4 days
Cryopreservation	In 70 % medium, 20 % FCS, 10 % DMSO
Morphology	medium-sized spheroidal morphologies
Viral status	Negative for EBV
Contamination	Negative for mycoplasma
Authentication	Yes (by DNA finger printing, cytogenetic characteristics, immunoprofile)
Immunoprofiles	
Myelocytic	CD13+, CD33+, CD15+, MPO+
B lymphoid	CD10+, CD19+, CD22+, CD23+, cCD79a+
Megakaryocytic	CD41+
Progenitor	CD38+, HLA-DR+
Plasma cell	CD138+
Genetic characterization	
Karyotypic analysis in conjunction with SKY	46,XX,del(2)(q33)t(2;2)(p22;q37), t(4;5)(q35;q35),t(5;8)(q32;q22), der(6)del(6)(p21p22)t(6;10)(p23;q23), t(7;21)(p15;q21,der(9)del(9)p21)del(9)(q34.2), der(10)t(6;10),t(12;22)(p13;q13), der(17)t(17;17)(p13;q22),del(19)(q13)
Array-CGH	del(2)(q33.1-q37.3), del(6)(p21.2-p21.31), del(8)(q21.2), del(8)(q23.3-q24.11), del(9)(q21.33-q34.12), del(10)(q23.33-q24.1), del(10)(q25.1), del(12)(p13.2), del(19)(q13.32), amp(17)(q21.32-q25.3)
Next-generation sequencing	
Whole-exome sequencing	Somatic mutations in ABCA8, BCHE, CALCA, CSTF2, FPR1, KCNJ8, MAFB, STMN1, TAAR8
Transcriptome sequencing	EP300-ZNF384, CHD4-EP300, MSH2-NLK, HACL1-COLQ, HDAC8-CITED1, POLA2-CDC42EP2

In summary, we established a novel MPAL cell line, JIH-5, and characterized its biologic background comprehensively to show a novel oncogenomic gene fusion together with an associated cluster of mutations. Our findings suggested that the JIH-5 cell line may serve as a tool for the study of MPAL or EP300-ZNF384.

## Abbreviations

B-ALL: B cell acute lymphoblastic leukemia
CNA: copy number alterations
EBV: Epstein-Barr virus
FCM: flow cytometry
FISH: fluorescence in situ hybridization
MOAP: mitoxantrone, cytosine arabinoside, vindesine, and dexamethasone
MPAL: mixed-phenotype acute leukemia
NGS: next-generation sequencing
NuRD: nucleosome remodeling and deacetylase
PCR: polymerase chain reaction
SKY: spectral karyotype
T-ALL: T cell acute lymphoblastic leukemia
